# St. Bartholomew's Hospital

**Published:** 1919-10-18

**Authors:** 


### The Hospital

The Workers' Newspaper of Administrative Medicine and Institutional Life, Administration, National Insurance and Health.

No. 1741, Vol. LXVII. SATURDAY, OCTOBER 18, 1919. PRICE TWOPENCE

### ST. BARTHOLOMEW'S HOSPITAL.

Everyone knows something at least of the history of St. Bartholomew's Hospital, one of the most ancient institutions in the land: older than Magna Charta, older than Parliament, older than the Lord Mayoralty of London, founded by Rahere in the reign of Henry the First, within the lifetime of some who had played a part in the Norman Conquest. Many know also, especially in the institutional world, that for several generations the hospital has been free, owing to the revenue derived from ancient endowments, from those pecuniary necessities and embarrassments which beset the managers of most voluntary hospitals. This fortunate state of affairs can now no longer be said to exist. St. Bartholomew's still has a large income, but the calls upon that income, and the response made to those calls, have entailed a steadily growing expenditure, which for some years has threatened to exceed the income. War conditions have accentuated these difficulties; and to-day the hospital is faced with a momentous crisis. Unless additional support is forthcoming, the work of the hospital will be perforce curtailed. For 1918 a deficit of £6,795 has been incurred, and the estimates for 1919 foreshadowed an additional deficit of £18,295, unless the income of the charity is supplemented to that extent from external sources. The rebuilding of the nurses' quarters, long overdue, has been postponed for lack of funds; and the convalescent home at Swanley has been closed down. These two pregnant and serious facts are enough of themselves to prove the reality of the straits in which the management stands financially. Both these needs will require very substantial sums to remove what can only be described as reproaches—reproaches not to St. Bartholomew's so much as to the public.

The cost of running the hospital for the year 1918 was over £122,000; towards which the subscription list provided only £11,309. This simple statement shows what a happy position St. Bartholomew's has hitherto stood in. The bulk of these subscribers, doubtless, are city firms and employers; for "Bart's" has always been peculiarly associated with the City of London. While, therefore, it is reasonable enough that the Committee should look first, and we feel sure not in vain, towards the City of London for an increase of the comparatively slender support which has hitherto alone been necessary, yet it is also quite right that in the changed circumstances and requirements of the day a wider basis of financial assistance should be solicited from the general public. For St. Bartholomew's, equally with the other great hospitals of the Metropolis, is a national possession, a national asset, and a national responsibility. It caters neither exclusively for the City, nor exclusively for London, nor exclusively for England: its patients are drawn from all over the Empire, and even from outside it. To inaugurate the special appeal now being issued, the Lord Mayor is providing a luncheon at the Mansion House on October 21, which, though it has necessarily to be limited in point of numbers, is to be made as representative and national in composition as possible. This official recognition is but the prelude to a widespread appeal, to which there is certain to be a generous response if those to whom it is addressed reflect for a moment on the history, the work, and the fame of this splendid and typically English institution, now almost eight centuries old.

To the Committee, the Treasurer, and the Organising Director of the appeal, we wish nothing but a splendid success, convinced that the institution deserves it and that it is inevitable provided skilful publicity is given to the needs and the work of St. Bartholomew's Hospital. With such a success duly attained, the institution must for its part rise to the situation, keep ever thoroughly abreast of the march of progress, and maintain a degree of all-round efficiency worthy of its wonderful history and of the great city which it is privileged to serve.

